# A study of differential microRNA expression profile in migraine: the microMIG exploratory study

**DOI:** 10.1186/s10194-023-01542-z

**Published:** 2023-02-17

**Authors:** V. J. Gallardo, J. B. Gómez-Galván, L. Asskour, M. Torres-Ferrús, A. Alpuente, E. Caronna, P. Pozo-Rosich

**Affiliations:** 1 Headache and Neurological Pain Research Group, Vall d’Hebron Institute of Research (VHIR), Universitat Autònoma de Barcelona, Barcelona, Spain; 2 Neurology Department, Headache Unit, Vall d’Hebron University Hospital, Barcelona, Spain

**Keywords:** Migraine, Epigenetics, Epigenetic mechanisms, microRNA, miRNA, Biomarkers, Personalized medicine

## Abstract

**Background:**

Several studies have described potential microRNA (miRNA) biomarkers associated with migraine, but studies are scarcely reproducible primarily due to the heterogeneous variability of participants. Increasing evidence shows that disease-related intrinsic factors together with lifestyle (environmental factors), influence epigenetic mechanisms and in turn, diseases. Hence, the main objective of this exploratory study was to find differentially expressed miRNAs (DE miRNA) in peripheral blood mononuclear cells (PBMC) of patients with migraine compared to healthy controls in a well-controlled homogeneous cohort of non-menopausal women.

**Methods:**

Patients diagnosed with migraine according to the International Classification of Headache Disorders (ICHD-3) and healthy controls without familial history of headache disorders were recruited. All participants completed a very thorough questionnaire and structured-interview in order to control for environmental factors. RNA was extracted from PBMC and a microarray system (GeneChip miRNA 4.1 Array chip, Affymetrix) was used to determine the miRNA profiles between study groups. Principal components analysis and hierarchical clustering analysis were performed to study samples distribution and random forest (RF) algorithms were computed for the classification task. To evaluate the stability of the results and the prediction error rate, a bootstrap (.632 + rule) was run through all the procedure. Finally, a functional enrichment analysis of selected targets was computed through protein–protein interaction networks.

**Results:**

After RF classification, three DE miRNA distinguished study groups in a very homogeneous female cohort, controlled by factors such as demographics (age and BMI), life-habits (physical activity, caffeine and alcohol consumptions), comorbidities and clinical features associated to the disease: miR-342-3p, miR-532-3p and miR-758-5p. Sixty-eight target genes were predicted which were linked mainly to enriched ion channels and signaling pathways, neurotransmitter and hormone homeostasis, infectious diseases and circadian entrainment.

**Conclusions:**

A 3-miRNA (miR-342-3p, miR-532-3p and miR-758-5p) novel signature has been found differentially expressed between controls and patients with migraine. Enrichment analysis showed that these pathways are closely associated with known migraine pathophysiology, which could lead to the first reliable epigenetic biomarker set. Further studies should be performed to validate these findings in a larger and more heterogeneous sample.

## Background

Migraine is a genetically-driven, chronic brain disease that ranks second on the most disabling neurological disorders of the Global Burden of Disease Study [1, 2]. If not adequately diagnosed and treated, migraine can increase in frequency and transform, through a chronification process, into chronic migraine (CM), which is defined by having 15 or more headache days per month [3], where patients’ burden increases exponentially [4]. Multiple factors, including both genetic and environmental mechanisms seem to play a role in migraine and its chronification [5–7] process, although the susceptibility of headache attack recurrence mainly depends on the brain-sensorial adaptability of both internal and external stimuli [8, 9]. Moreover, migraine stills represent a clinical challenge because, up to date, disease diagnosis, evolution and treatment response cannot be predicted due to the lack of reliable biomarkers.

From large-scale genome-wide association studies (GWAS) performed in the last decades, we have learnt that migraine has a polygenic heritability pattern and, up to now, we have identified more than 120 risk loci associated to migraine [10], mainly codifying for neuronal and vascular functions. However, the effect size of each individual reported gene is relatively modest and most of these genes have regulatory effects on gene expression rather than protein coding. Environmental factors may also modulate the expression of DNA. Therefore, the study of epigenetics which are the environmental influenced genetic regulatory mechanisms [11] could provide further understanding on migraine pathophysiology as well as, give insight to the dynamic aspect of the disease specifically contributing to its chronification [12].

Among the epigenetic regulatory mechanisms, microRNAs (miRNA) are a subtype of noncoding RNA of ∼22 nucleotides long that act as key regulators of genetic expression by inhibiting transcription or promoting degradation of selected messenger RNAs (mRNAs). A single miRNA sequence could regulate multiple mRNA, exerting a pleiotropic modulation of cellular processes. miRNA have been found intracellularly and in all body fluids, including plasma, saliva and urine [13]. Accessing the central nervous system (CNS) is too invasive and there is an increasing evidence that a relationship between peripheral blood mononuclear cells (PBMC) and brain-based epigenetic activity exists [14]. Thus, PBMC gene expression may reflect both central and peripheral neurological mechanisms in migraine, induced, for example in migraine by the activation and neuroinflammation of the trigeminocervical pain pathway [15]. In fact, previous studies in headache disorders have shown that transcriptional changes of PBMC can reflect alterations in the sensitization of the peripheral and/or central pain pathway [16, 17].

Several pilot studies have described miRNAs as potential peripheral biomarkers of migraine disease [18–26]. Nevertheless, studies are scarcely comparable and reproducible not only because of the lack of standardized protocols, biological matrix or sample size but mainly because of the heterogeneous population and clinical variability included. Increasing evidence shows that disease-related intrinsic factors altogether with lifestyle factors may influence epigenetic mechanisms and identifying them is crucial to find unbiased reliable factors associated with a specific disease [27–29].

Hence, the goal of this exploratory study is to find differentially expressed miRNAs (DE miRNA) in PBMC of patients with migraine compared to healthy controls in a well-controlled homogeneous non-menopausal female cohort.

## Methods

### Participants and protocol approval

The study was approved by our Institutional Review Board (PR(IR)224/2016). All participants provided written informed consent before participation. We recruited patients treated for migraine in the Headache Outpatient Clinic. Patients had a diagnosis of migraine with or without aura, confirmed by a neurologist according to the International Classification of Headache Disorders, 3rd edition (ICHD-3) [3]. Healthy controls (HC) were recruited among hospital staff and non-related acquaintances of patients who denied past or first-degree familial history of any recurrent primary or secondary headache disorder. To diminish confounding factors, the inclusion criteria were: female sex, 18–45 years old (non-menopausal) and Mediterranean ethnicity for all participants. Moreover, patients could not have received prophylactic treatment for migraine prevention or if they received it, it had been done for a brief period of time more than one year or more before the study and without following national guidelines of effective dosage and duration. Due to the strict inclusion criteria, participants were included from August 2016 to September 2017.

As miRNAs can be modulated by different lifestyle habits and disease characteristics, recruited participants completed a very thorough and complete questionnaire and structured-interview to collect demographic information including: age, sex, ethnicity, educational level, marital status, gross income; clinical information including: habits of caffeine, tobacco and drugs consumption, physical activity level using the International Physical Activity Questionnaire (IPAQ-SF) [30]; life-time comorbidities, medications use, menstrual status and migraine information including disease duration, acute medication use, monthly headache frequency and family history of headache. We registered headache pain, accompanying symptoms and menstrual cycle the day of the blood sample extraction. We only analyzed samples from migraine patients with at least 48 h of a headache-free period prior day of the blood extraction. In addition, all patients had to fill in the following scales: Allodynia Symptom Checklist (ASC-12) [31], Headache Impact Test-6 (HIT-6) [32], Migraine Disability Assessment Test (MIDAS) [33], Beck Depression Inventory 2^nd^ Edition (BDI-II) [34], State-Trait Anxiety Inventory (STAI) [35] and Perceived stress scale (PSS) [36]. Patients were asked to complete headache diary 30 days before extraction to confirm migraine diagnosis (screening period). We also excluded patients who fulfilled acute medication overuse headache criteria during the screening period.

### Blood extraction, PBMC isolation and RNA extraction

For blood extraction patients were asked to fast for 6 h, minimize their exercise/activity and not take analgesic medication 24 h prior to blood extraction. Peripheral blood was collected by antebrachial vein puncture using three, 8 mL, BD Vacutainer® CPT™ Cell Preparation Tubes with Sodium Citrate (BD, Franklin Lakes, NJ, USA) and processed within 2 h following the manufacturer’s instruction to isolate PBMC pellets that were immediately stored at -80ºC till miRNA extraction. To minimize variability due to manipulation, the miRNA extraction was made by the same laboratory technician in the same facilities. For miRNA extraction, we used the Qiagen miRNeasy Mini Kit (Qiagen, Valencia, CA, USA), following the manufacturer’s instruction. RNA concentration was measured using Nanodrop 2000 (NanoDrop Technologies, Wilmington, DE). RNA integrity number (RIN) was determined the kit Agilent RNA 6000 Nano by the Bioanalyzer Agilent 2100 (Agilent Technologies, Santa Clara, CA, USA). We excluded from analysis samples with RIN under 6.

### Microarray hybridization

Microarrays service was outsourced and carried out in at High Technology Unit at Vall d’Hebron Research Institute (HTU-VHIR), Barcelona (Spain). For this study, we used the Affymetrix GeneTitan microarray platform and the Genechip miRNA array plate 4.1. Starting material was 550 ng of total RNA of each sample. MiRNA in the sample was labelled using Flash Tag Biotin HSR RNA Labelling kit following the manufacturer’s instructions.

### Statistical analysis

All statistical analysis were conducted in R v4.2.0 [37] and figures were produced using the package ggplot2 v.3.3.6 [38]. A statistical power calculation was not conducted prior to the study because the sample size was based on the available data for this exploratory analysis. However, to increase the likelihood of finding differential miRNA expression profiles for this study, we selected participants from a well-controlled homogeneous non-menopausal female cohort. *P*-values < 0.05 were considered as statistically significant and are reported for a two-tailed test.

#### Clinical analysis

Nominal variables (aura and presence of allodynia) were reported as frequencies (percentages) while median and interquartile range (IQR) were reported for quantitative variables: age, BMI, IPAQ-SF, caffeine and alcohol consumptions, STAI, BDI-II, PSS, disease evolution time, ASC-12, monthly headache frequency (MHD), monthly acute medication intake (MAMI), MIDAS and HIT-6. Normality assumption of quantitative variables was checked through visual methods (Q-Q plots) and normality tests (Shapiro–Wilk test). Statistical significance between study groups (HC vs. patients with migraine) was assessed by unpaired t-test for BMI, caffeine consumption, STAI and PSS was used and unpaired Wilcoxon rank-sum test was used for the other quantitative variables that did not follow any normality assumption.

#### Bioinformatic analysis

The statistical analysis of the Genechip miRNA array plate 4.1 was performed using libraries developed for the microarray analysis in the Bioconductor Project [39]. At the moment of the analysis, there were no specific packages for miRNA 4.1 arrays available, thus we programmed our own package. Quality control (QA) was assessed with the package arrayQualityMetrics before the normalization process [40]. Additionally, principal components analysis (PCA) and hierarchical clustering analysis (HCA) were performed to study samples distribution and detect technical problems, including batch effect. Each array (CEL file) was preprocessed and background corrected, normalized and summarized using RMA (Robust Multiarray Average) through Bioconductor packages [41, 42].

The identification of differentially expressed miRNA (DE miRNAs) was based on adjusting linear model with empirical Bayes moderated t-statistic, conducted by the limma package [43] in the comparison between controls and patients with migraine. Models were adjusted by covariates using surrogate variables estimation [44]. DE miRNAs that had more than 0.5-fold increase/decrease relative to controls were chosen for further analysis. The minimum of 0.5-fold expression difference cut-off has been chosen based on the several miRNA profiling studies using microarrays; confirming that subtle changes in miRNA expression, such as a 0.5-fold difference, have a significant biological impact [45–48]. Results were further corrected for multi-testing using the Benjamini–Hochberg procedure for control the false discovery rate (FDR) [49]. Features that met both criteria (adjusted *t*-test and ± 0.5 fold change) were combined in a single matrix of DE miRNAs that underwent classification analysis.

We used Random Forests (RF) models for the classification task, a machine learning method widely used in the microarray analysis [50–52]. RF algorithm uses an ensemble of classification trees, internally and randomly constructed using a bootstrap sample of the data. Then, the algorithm merges all constructed tree altogether (forest) in order to get an accurate and stable prediction [53, 54]. Once we obtained the forest, the construction of the classifier is performed by a feature reduction algorithm, where the less important features are successively eliminated and out of the bag (OOB) error is continuously analyzed. To evaluate the stability of the results and the prediction error rate, a bootstrap (0.632 + rule) was run through all the procedure. All classification task and bootstrap were performed through the varSelRF R package [55, 56].

Target genes of selected DE miRNAs were predicted by using the miRDB database through the multimiR package [57] and functional and pathway enrichment analysis was performed using the pathfindR package [58]. PathfindR identifies gene sets that form active subnetworks in a protein–protein interaction network (PIN) in order to identify distinct active subnetworks and then perform enrichment analyses on these subnetworks. For assigning a significance value for each predicted target gene (pathfindR input), all *Homo sapiens* miRNA-target gene predictions were obtained: 1,954,039 miRNA-gene predictions from all *H. sapiens* miRNAs listed in the miRDB (2,656 miRNAs). Then, the significance for each predicted gene was computed as the probability of observing a score greater than or equal to the score of this target gene over all *H. sapiens* miRNA-target gene scores [59]. For genes that were targeted by more than one miRNA, the lowest significance was kept.

## Results

### Clinical characteristics of the patients

**Fig. 1:**
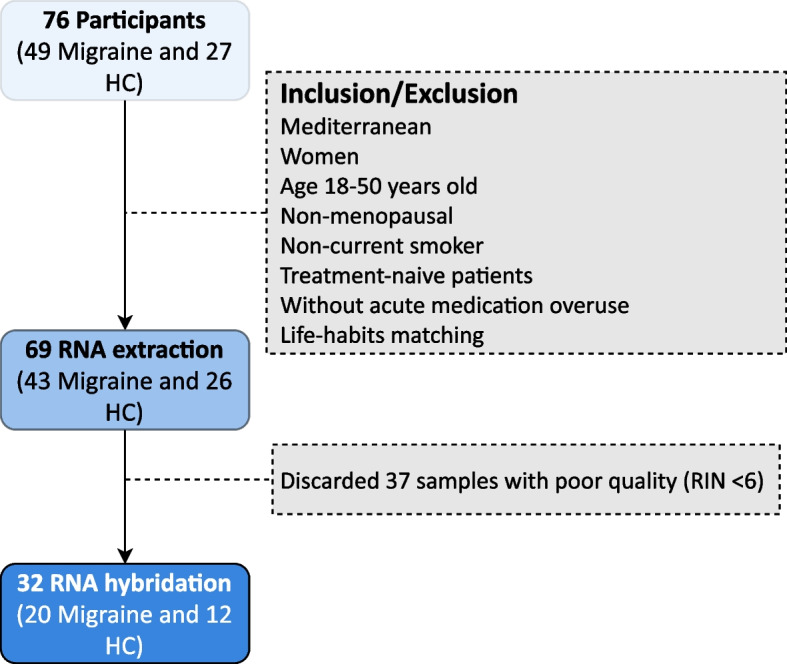
Patients’ recruitment flowchart. We recruited participants from August 2016 to September 2017. HC: healthy controls; RIN: RNA integrity number; QA: quality assessment

Participant recruitment is represented in Fig. 1. Since August 2016, a total of 76 (49 patients with migraine and 27 HC) participants were recruited. For this exploratory study, RNA was extracted from 69 samples that fulfilled inclusion/exclusion criteria. Among these, 37 showed a RIN < 6 and were discarded. Therefore, 32 participants (12 HC, 20 patients) were considered for the analysis with a median age of 33.2 [28.0, 39.8] years old. No statistically significant differences were found neither in demographical variables nor life-habits measurements between groups. In terms of comorbidities, we only found that patients with migraine presented a statistically significantly greater trait-anxiety score (HC: 31.0 [27.0, 38.2] vs. migraine: 39.5 [37.2, 55.8]; *p* = 0.007). In patients, median MHD was 10.5 [8.0, 24.2] days/month (d/mo) and median MAMI was 7.5 [4.0, 9.0] d/mo. Main demographic and clinical characteristics are shown in Table 1.

**Table 1:** Demographics, life habits, comorbidities and migraine characteristics

	**HC** (*n* = 12)	**Migraine** (*n* = 18)	*P* value
**Demographics**			
Age, years	30.0 [28.0, 33.5]	36.0 [28.2, 41.0]	0.319^**‡**^
Body mass index, kg/m^2^	22.7 [21.7, 23.2]	22.9 [20.4, 25.5]	0.594^†^
**Life-habits**			
IPAQ-SF, METs/week	3.1 [2.2, 4.2]	3.4 [1.4, 6.2]	0.750^**‡**^
Caffeine consumption, g/week	1.1 [0.9, 1.6]	1.0 [0.2, 1.4]	0.261^†^
Alcohol consumption, g/week	15.0 [0.0, 40.0]	0.0 [0.0, 10.0]	0.062^**‡**^
**Comorbidities**			
Anxiety (STAI), score			
State	35.0 [32.0, 41.0]	38.5 [36.2, 52.2]	0.075^†^
Trait	31.0 [27.0, 38.2]	39.5 [37.2, 55.8]	**0.007**^†^
Depression (BDI-II), score	4.0 [0.8, 6.2]	8.5 [1.2, 14.8]	0.232^**‡**^
Perceived stress (PSS), score	16.0 [12.8, 22.5]	19.5 [17.0, 32.0]	0.270^†^
**Migraine characteristics**			
Duration of migraine disease, y		19.5 [11.0, 27.0]	
Aura		38.9% (7)	
Allodynia		72.2% (13)	
ASC-12, score		4.5 [2.2, 6.0]	
Monthly headache frequency, d/mo		10.5 [8.0, 24.2]	
Monthly acute medication intake, d/mo		7.5 [4.0, 9.0]	
**Migraine-related clinical burden**			
Disability (MIDAS), score		28.5 [17.2, 47.8]	
Headache-related impact (HIT-6), score		63.5 [61.2, 69.0]	

Continuous data is represented in median [IQR] and categorical data in % (n).

**Bold** font indicates statistically significant variables

*IQR*: interquartile range, *HC* healthy controls, *y* years, *IPAQ-SF* short-form of the international physical activity questionnaire, *MET* metabolic equivalents of task, *STAI* State-Trait Anxiety Inventory, *BDI-II* Beck depression inventory-second edition, *PSS* perceived stress scale, *ASC-12* 12-item allodynia symptom checklist; *d/mo* days/month, *MIDAS* migraine disability assessment, *HIT-6* headache impact test

^†^Statistical significance assessed with unpaired t-test

^**‡**^Statistical significance assessed with unpaired Wilcoxon rank-sum test

No statistically significant differences were found in terms of RNA quality (RIN, median [IQR]) between study groups: 6.9 [6.5, 7.8] HC vs. 6.7 [6.3, 7.2] patients; *p* = 0.420.

### MiRNA microarray expression profiling

**Fig. 2:**
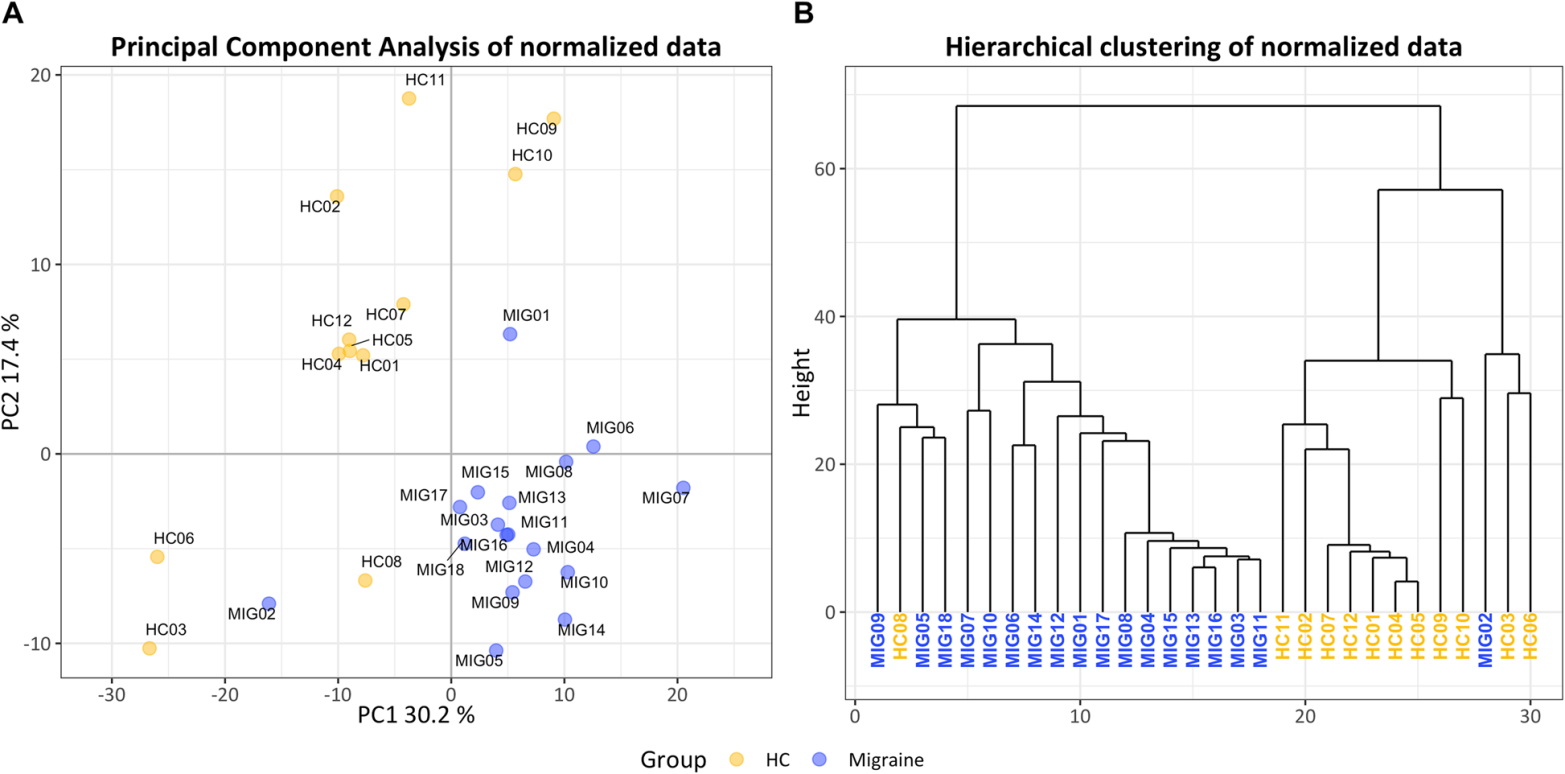
Principal component analysis (**A**) and unsupervised hierarchical clustering (**B**) of miRNA normalized expression data. Unsupervised hierarchical clustering (**B**) was computed through euclidian distances between samples. HC: healthy controls; MIG: patients with migraine, PC: principal component

During microarrays QA, we distinguished two samples (from patients) with higher values of intensity in all their probe sets. Similarly, normalized unscaled standard errors values and relative log expression values for the same two samples diverged from the rest, indicating aberrant expression. Hence, we discarded these 2 samples from further analysis (Fig. 1). Control and migraine samples were perfectly segregated along PCA and HCA (Fig. 2).

**Fig. 3:**
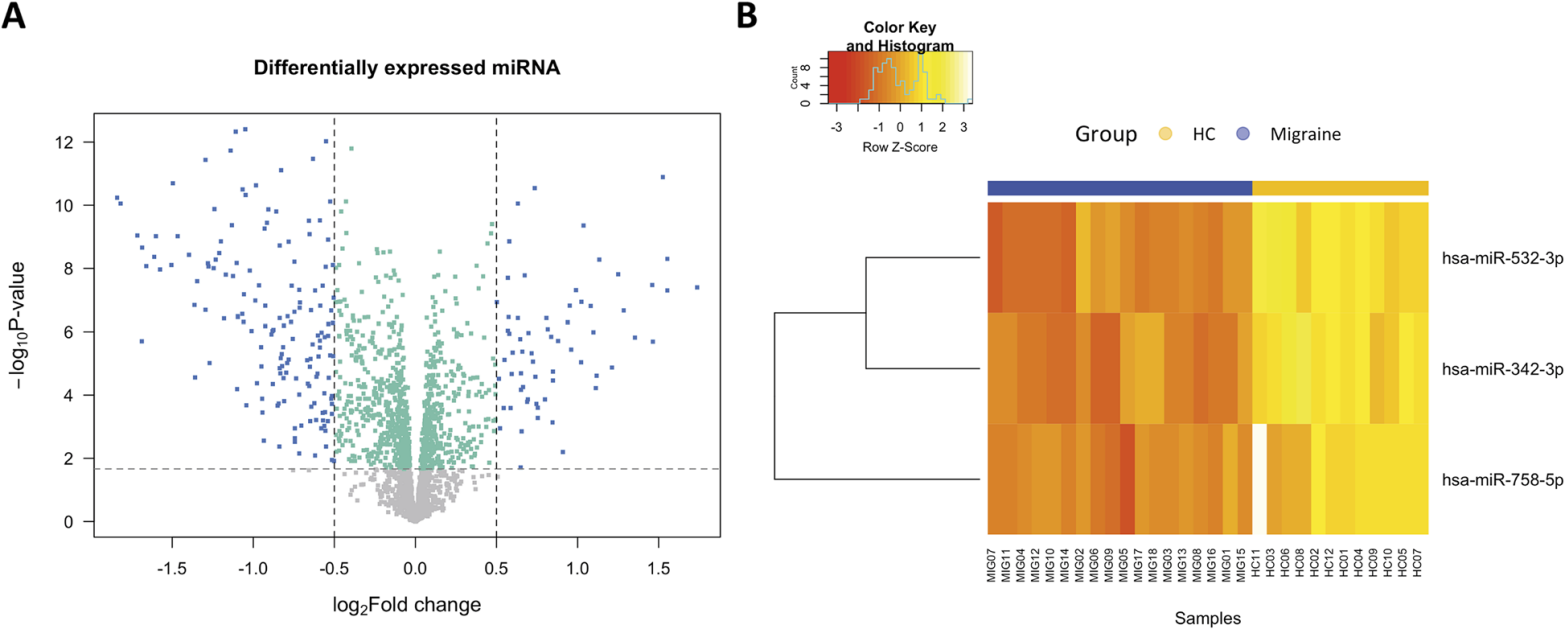
Volcano plot (**A**) and heatmap (**B**) of differentially expressed miRNA between healthy controls and patients with migraine. Scattered points from the volcano plot (**A**) represent miRNAs: the x-axis is the log twofold-change for the ratio healthy controls vs patients with migraine, whereas the y-axis is the log 10 *P*-value. Colored dots (blue and green) are differentially miRNA statistically significantly over- (right) and under-expressed (left) in patients compared to controls. Blue dots are differentially expressed miRNA with a log twofold-change > 0.50. A heatmap (**B**) of the 3-miRNA signature selected from the classification algorithm (RF). Columns represent individual arrays, while rows represent specific DE miRNA. The Z-score depicts a measure of distance, in standard deviations, away from the mean. The relative value for each miRNA is depicted by color intensity, with yellow indicating over-expression and red indicating under-expression. HC: healthy controls; MIG: patients with migraine

Adjusting by STAI-trait values, we found 191 DE miRNA between study groups: 59 over-expressed and 132 under-expressed transcripts in patients (Fig. 3A). Then, RF analysis combined with a feature selecting algorithm was used to build the classifier and to identify the best targets that distinguished between controls and migraine. The OOB for the initial model was 0.067 (91.7% sensitivity, 94.4% specificity) and a 3-miRNA signature was found as best variables without dropping the OBB error substantially: miR-342-3p, miR-532-3p and miR-758-5p. Then, as RF algorithms are deterministic, performing it on different training samples is the only way to generate diversity in the selection. To this end, we performed bootstrapping (+ 0.632) in which the random forest constructed for a certain number of variables was subsampled and compared. The prediction error rate among the bootstrap samples was 0.142. The same 3-miRNA signature were consistently selected (stability) in all the sub-samples, with miR-532-3p being selected in the 60% of cases. Figure 3B shows the heatmap from the selected DE miRNA between groups where the expression from all of them were under-expressed in patients: miR-342-3p (log_2_FC: -0.551), miR-532-3p (log_2_FC: -1.109) and miR-758-5p (log_2_FC: -0.508).

### Targets prediction and functional enrichment analysis

Predicted target genes associated with the 3-miRNA signature were retrieved from the miRBD database through the multimiR package. Each target significance was computed according to the prediction score retrieved from the miRDB (see Methods section). Then, a total of 68 target genes were identified: 42 genes from miR-342-3p, 26 genes from miR-532-3p and 9 genes from miR-758-5p. While four common target genes were found between miR-342-3p and miR-532-3p: ATXN7 (ataxin 7), CDKL5 (cyclin dependent kinase like 5), PAPPA (Pappalysin 1) and RRM2 (Ribonucleotide Reductase Regulatory Subunit M2); two common targets were found between miR-342-3p, miR-758-5p: DHRSX (Dehydrogenase/Reductase X-Linked), NUFIP2 (Nuclear FMR1 Interacting Protein 2); and 3 common targets between miR-532-3p and miR-758-5p: DSG3 (Desmoglein 3), PTP4A1 (Protein Tyrosine Phosphatase 4A1), TET3 (Tet Methylcytosine Dioxygenase 3). No common target gene was found for all three miRNAs.

Functional enrichment analysis of miRNA-target genes predicted from the miRDB was performed through active-subnetwork-oriented analysis via pathfindR and a total of 24 enriched KEGG pathways were identified (Table 2). Top pathway was calcium signaling pathway and a total of 9 different target genes were involve in all significantly enriched pathways: CACNA1C, HTR2C, EP300, PTGER4, ID4, RICTOR and EEA1 from miR-342-3p; PRKCA and DTX4 from miR-532-3p and ID2 from miR-758-5p.

**Table 2:** Enriched KEGG pathways of predicted target from the 3-miRNA differentially expressed signature

ID	KEGG Pathway	Adj. *p*-value	Target genes
hsa04020	Calcium signaling pathway	< 0.0001	CACNA1C, HTR2C, PRKCA
hsa04330	Notch signaling pathway	0.001	DTX4, EP300
hsa04750	Inflammatory mediator regulation of TRP channels	0.002	HTR2C, PTGER4, PRKCA
hsa04720	Long-term potentiation	0.002	CACNA1C, EP300, PRKCA
hsa05031	Amphetamine addiction	0.003	CACNA1C, PRKCA
hsa04540	Gap junction	0.005	HTR2C, PRKCA
hsa04350	TGF-beta signaling pathway	0.006	ID2, ID4, EP300
hsa04912	GnRH signaling pathway	0.006	PRKCA, CACNA1C
hsa04713	Circadian entrainment	0.007	CACNA1C, PRKCA
hsa04925	Aldosterone synthesis and secretion	0.007	PRKCA, CACNA1C
hsa04916	Melanogenesis	0.008	EP300, PRKCA
hsa04066	HIF-1 signaling pathway	0.010	EP300, PRKCA
hsa04726	Serotonergic synapse	0.011	CACNA1C, HTR2C, PRKCA
hsa04935	Growth hormone synthesis, secretion and action	0.013	EP300, PRKCA, CACNA1C
hsa04919	Thyroid hormone signaling pathway	0.014	EP300, PRKCA
hsa04270	Vascular smooth muscle contraction	0.015	CACNA1C, PRKCA
hsa04728	Dopaminergic synapse	0.018	PRKCA, CACNA1C
hsa04261	Adrenergic signaling in cardiomyocytes	0.025	CACNA1C, PRKCA
hsa04921	Oxytocin signaling pathway	0.027	PRKCA, CACNA1C
hsa04150	mTOR signaling pathway	0.028	RICTOR, PRKCA
hsa05161	Hepatitis B	0.033	EP300, PRKCA
hsa04310	Wnt signaling pathway	0.034	EP300, PRKCA
hsa05164	Influenza A	0.039	EP300, PRKCA
hsa05152	Tuberculosis	0.046	EP300, EEA1

Adj. *p*-value are the lowest enrichment *p*-value obtained through multiple iterations. The target genes are the predicted genes (targets of at least one DE miRNA) involved in the given enriched pathway

*KEGG* Kyoto Encyclopedia of Genes and Genomes, *Adj. p-value* adjusted p-value corrected for multi-testing using the Benjamini–Hochberg procedure (FDR)

## Discussion

In this exploratory study, we found 191 differentially expressed miRNAs (59 over-expressed and 132 under-expressed transcripts) between controls and patients with migraine in a very homogeneous female cohort, controlled by factors as demographics, life-habits and comorbidities (Table 1, Fig. 3A). After transcripts classification task, we finally selected three DE miRNAs that distinguished study groups: miR-342-3p, miR-532-3p and miR-758-5p. All three DE miRNAs were under-expressed in patients compared to controls (Fig. 3B). Sixty-eight target genes were predicted from this 3-miRNA signature and in the functional analysis, we found that they mainly enriched ion channels and signaling pathways (calcium, notch, TRP, TGF-beta, GnRH, HIF-1, thyroid, oxytocin, mTOR and Wnt), neurotransmitter and hormone homeostasis (aldosterone, serotonin, growth hormone, dopamine and adrenaline), infectious diseases (Hepatitis, Influenza and tuberculosis) and circadian entrainment (Table 2).

MiRNAs may have the potential to accurately diagnose disorders at an early stage. For instance, it has been found that levels of miR-182 in the peripheral blood of patients with gliomas increase with disease grade, which had allowed to researchers to patent a diagnostic kit for early diagnosis of human glioma (patent number: CN101792793A) [60]. This is beneficial for patients in order to receive earlier and more individual targeted treatment therapies, a milestone in migraine since we already know that if it is not adequately diagnosed and treated, migraine frequency increases until disease chronification [61]. Moreover, there is increasing evidence that the later we start treating migraine, less effective is the treatment and more difficult it becomes to reverse the disease [5, 62]. In regards to CNS diseases, specific DE miRNAs have also been identified and validated in order to diagnose multiple sclerosis (US9187785B2) [63], Alzheimer’s disease (EP2733220A1) [64, 65] or major depressive disorder (EP2529222A4) [66]. MiRNAs have also been given considerable attention as a therapeutic targets/agent. In recent years, miRNA-based therapeutic approaches have been developed in CNS diseases, especially pre-clinical studies in neurodegenerative disorders [67], although clinical trials have not started yet [68]. Hence, these patents and previous studies in other diseases highlight that investigating miRNA profiles in migraine will lead us not only to a better understanding of their pathogenesis, but also to help develop diagnostic kits, identify specific cluster of patients, explore disease onset, monitor migraine attacks, personalize therapeutic treatment and revert disease chronification.

Several authors have made an effort in previous studies to identify potential miRNAs as peripheral biomarkers of migraine [12]. Numerous miRNAs have been described associated with pain-free patients with migraine in comparison to healthy controls: miR-382-5p (over-expressed in serum and PBMC) [21, 23]; miR-34a-5p (over-expressed in serum, saliva and PBMC) [21, 24]; miR-27b (over-expressed in plasma) [25]; miR-181a, let-7b, and miR-22 (under-expressed in PBMC) [25]; miR-155, miR-126, and let-7 g (over-expressed in plasma) [18]; miR-30 (over-expressed in PBMC) [69]; miR-5189-3p, miR-3613-5p, miR-99a-3p, miR-542-3p (over-expressed in PBMC) and miR-96-5p (under-expressed) [26]. Results are heterogenous and study-dependent but not only because there are differences in lab procedures, biological matrix or sample size but mainly because of the large degree of heterogeneity between study groups. Published studies only controlled by demographic factors (age and sex) but did not consider neither participants selection nor analysis with an adjustment by covariates other factors such as comorbidities and lifestyle habits. Epigenetics have been established as a molecular link between genetic function and the environment. Hence, epigenetic marks constitute a malleable language by which internal and external factors can influence the genome and, for that reason, it is crucial to be able to separate correlative from causal changes in order to understand and quantify the effect of the epigenetic function in diseases [70]. Nowadays, we have more evidence that lifestyle factors such as diet [71], smoking [72], alcohol [73, 74], stress [75], physical activity [29, 76], treatments [77–79] and comorbidities [80, 81] have a great impact on the dynamisms of epigenetic marks. Controlling them will allow us to identify transcripts associated with a disease that are not unmasked for other factors.

Functional analysis showed that targeted genes associated to the 3-miRNA signature enriched pathways have been previously described and associated with migraine pathophysiology. One of the most enriched targets is CACNA1C which encodes for the alpha-1 subunit of a voltage-dependent calcium channel. Calcium channels mediate the influx of calcium ions into the cell upon membrane polarization and they are encoded by different subunits of the CACNA1 gene (CACNA1A-S) [82, 83]. From migraine pathophysiology, we already know that the trigeminovascular system (TVS) can be activated by cortical spreading depression (CSD). Mutations in CACNA1A can disrupt ionic homeostasis, increasing the susceptibility to CSD and inducing migraine attacks [84]. Moreover, a CACNA1A variant has also been associated with migraine with aura in the last migraine association study [10]. So, a down-regulation of CACNA1C gene could be also associated with these ionic disturbances in neuronal synapses; indicating that miR-342-3p has a reliable effect on migraine pathophysiology.

Other enriched pathways codifying for vascular functions which may have and important role in migraine etiopathogenesis were the notch signaling pathway, the inflammatory mediator regulation of TRP channels and the HIF-1 signaling pathway. Notch receptors (Notch1-4) are a family of type-1 transmembrane proteins which are essential for embryonic development. Mutations in the Notch pathway genes, like NOTCH2 and JAG1, are associated with vascular abnormalities [85]. In migraine, there is evidence that distinct polymorphisms of NOTCH4 gene significantly modify clinical characteristics of migraine phenotype [86]. The Transient Receptor Potential (TRP) channels are a superfamily of integral membrane proteins well-described in pain conditions that functions as cation channels: TRPA, TRPC, TRPM, TRPP, TRPL, and TRPV [87–89]. These channels contribute to a several physiological processes ranging from thermosensation and pain to the regulation of Ca2 + levels in the endoplasmic reticulum. TRP channels are also expressed in the meningeal nociceptors and it has been proved that their activation, promotes the release of the neuropeptide calcitonin gene related peptide (CGRP) [90]. The role of CGRP in migraine has been exhaustively described in both preclinical and clinical studies during the last decades [91] and the CGRP antagonism has shown to be efficacious for the treatment of migraine [92–94]. However, the mechanism by which CGRP is released during migraine is unclear, and for that reason, TRP channels remain a focus of interest for their potential contribution to attacks [95]. Moreover, from our experience in clinical trials and real-world studies there are patients with migraine who do not respond to treatments, even target-driven one such as anti-CGRP therapies [96], suggesting that CGRP is not the only mechanism driving migraine attacks [97, 98]. In regards to hypoxia-inducible factor 1 (HIF-1), it is a transcription factor that functions as a master regulator of oxygen homeostasis. Since HIF-1α is implicated in neuroprotection and inflammation inhibition, a recent study has demonstrated that roxadustat administration, an anti-anemia medication, ameliorated migraine-like behaviors and inhibited central pain sensitization in nitroglycerin-injected mice, which was mainly mediated by HIF-1α/NF-κB/inflammation pathway [99].

The relationship between hormones and neurotransmitters with migraine is not entirely clear, but we do know it exists and plays a fundamental role in its pathophysiology. On one hand, thyroid hormones, along with PTX3 and, insulin resistance share common mechanisms in the development of endothelial dysfunction [100], a vascular pathophysiological genetically driven mechanism of migraine [101]. Moreover, differentially pattern levels have been found between controls and patients with migraine of the hypothalamic-tuberoinfundibular system (prolactin and growth hormone) [102]. On the other hand, the gonadotropin releasing hormone (GnRH) controls the release of sexual hormones although estrogens can negatively regulate the GnRH from the hypothalamus at the time of the preovulatory surge, suggesting that estrogen could be related to the increase in migraine prevalence in relation with puberty and ovulation in women [103, 104]. Other hypothalamic hormone linked to migraine is the Oxytocin (OT). OT has been used to relieve migraine attacks through intranasal or intravenous administration [105] since OT receptors included in the trigeminal ganglia and neurons can inhibit the release of CGRP [106].

Altogether, the 3-miRNA signature found could be a reliable biomarker for migraine diagnosis not only because they are differentially expressed between female patients and controls, but they are also involved in enriched biological processes which have been previously described in migraine pathophysiology. So, the under-expression of these transcripts may cause a dysregulated expression of genes associated with the vascular dysfunction and neurohormonal imbalances characterized by patients with migraine.

This study has limitations. First, RNA-seq is the most commonly used NGS technique to explore differences at transcript level to date. RNA-seq is able to detect novel transcripts with higher specificity and sensitivity. However, at the moment of designing and planning the current study, arrays where the more standardized technology. In addition, the RNA-Seq procedure has a few disadvantages compared to arrays: a lack of optimized and standardized protocols for analysis [107, 108]; the size of RNA-Seq files is considerably larger; and RNA-seq requires highly intensive and expensive computation infrastructure and analytics, as well as, longer analysis times [108, 109]. Nevertheless, arrays technologies are still a reliable technology to study gene expression with results comparable to RNA-seq [110]. Second, we did not evaluate the hypothesis that the miRNA profile could be related to the phenotype of circulating PBMC phenotype, rather than a differential miRNA expression. Several changes in PBMC profile related to migraine have been reported but, as different methodologies were used, the overall results are inconclusive. For instance, a meta-analysis of studies from 1966 to 1999 addressing immunological changes related to migraine did not find clear alterations in circulating leukocytes or other immunological parameters [111]. More recently, a relative lymphocytosis has been reported in chronic migraine associated with medication overuse headache, that was considered probably related to a chronic inflammatory state and stress response rather than specifically to migraine [112, 113]. Finally, the sample was small, and highly selected and homogeneous, only considering non-menopausal women in order to reduce sample size and confounding factors. Moreover, the group of individuals diagnosed with migraine was not totally homogeneous in terms of disease pathophysiology (e.g., either episodic or chronic migraine and either the presence or absence of aura). Hence, this 3-miRNA signature should be validated in a larger sample size and more heterogeneous cohort. However, data gathered in this exploratory study will be used to calculate the required sample size for the subsequent discovery and validation (RT-qPCR) studies. Due to lack of power in small sample size experiments, true effects are often missed and many of the detected effects will probably not be further validated. Therefore, the objective of this study was to to perform an exploratory study using the same in- and exclusion criteria for a miRNA high-throughput screen [114].

## Conclusion

A 3-miRNA signature (miR-342-3p, miR-532-3p and miR-758-5p) has been found differentially expressed between controls and patients with migraine in a very homogeneous cohort of non-menopausal women, controlled by factors such as demographics, life-habits and comorbidities. Enrichment analysis show that these three miRNAs are closely associated with migraine pathophysiology, maybe becoming the first reliable biomarker of disease. Further studies will be performed to validate them in a larger and more heterogeneous sample.

## Abbreviations

ASC-12: Allodynia Symptom Checklist
BDI-II: Beck Depression Inventory 2nd Edition
CM: Chronic Migraine
CNS: Central Nervous System
DE: Differentially Expressed
FDR: False Discovery Rate
GWAS: Genome-Wide Association Studies
HC: Healthy Controls
HCA: Hierarchical Clustering Analysis
HIT-6: Headache Impact Test-6
ICHD-3: International Classification of Headache Disorders (3rd edition)
IQR: Interquartile Range
IPAQ-SF: International Physical Activity Questionnaire (Short Form)
MAMI: Monthly Acute Medication Intake
MHD: Monthly headache frequency
MIDAS: Migraine Disability Assessment Test
miRNA: MicroRNA
mRNA: Messenger RNA
OOB: Out Of the Bag
PBMC: Peripheral Blood Mononuclear Cells
PCA: Principal Components Analysis
PIN: Protein-protein Interaction Network
PSS: Perceived Stress Scale
QA: Quality Assessment
RF: Random Forest
RIN: RNA Integrity Number
RMA: Robust Multiarray Average
STAI: State-Trait Anxiety Inventory
VHIR: Vall d’Hebron Research Institute

## References

[1] Stovner LJ, Nichols E, Steiner TJ, Abd-Allah F, Abdelalim A, Al-Raddadi RM (2018). Global, regional, and national burden of migraine and tension-type headache, 1990–2016: a systematic analysis for the Global Burden of Disease Study 2016. Lancet Neurol.

[2] Stovner LJ, Hagen K, Linde M, Steiner TJ (2022). The global prevalence of headache: an update, with analysis of the influences of methodological factors on prevalence estimates. J Headache Pain.

[3] Headache Classification Committee of the International Headache Society (2018). The International Classification of Headache Disorders, 3^rd^ edition. Cephalalgia.

[4] Lanteri-Minet M (2014). Economic burden and costs of chronic migraine. Curr Pain Headache Rep.

[5] May A, Schulte LH (2016). Chronic migraine: risk factors, mechanisms and treatment. Nat Rev Neurol.

[6] Goadsby PJ, Charbit AR, Andreou AP, Akerman S, Holland PR (2009). Neurobiology of migraine. Neuroscience.

[7] Torres-Ferrús M, Ursitti F, Alpuente A, Brunello F, Chiappino D, de Vries T (2020). From transformation to chronification of migraine: pathophysiological and clinical aspects. J Headache Pain.

[8] Bernstein C, Burstein R (2012). Sensitization of the trigeminovascular pathway: perspective and implications to migraine pathophysiology. J Clin Neurol.

[9] Schwedt TJ (2013). Multisensory integration in migraine. Curr Opin Neurol.

[10] Hautakangas H, Winsvold BS, Ruotsalainen SE, Bjornsdottir G, Harder AVE, Kogelman LJA, et al (2022) Genome-wide analysis of 102,084 migraine cases identifies 123 risk loci and subtype-specific risk alleles. Nat Genet 54(2):152–60 10.1038/s41588-021-00990-0 PMC8837554 35115687

[11] Tammen SA, Friso S, Choi SW (2013). Epigenetics: The link between nature and nurture. Mol Aspects Med.

[12] Gallardo VJ, Vila‐Pueyo M, Pozo‐Rosich P (2023) The impact of epigenetic mechanisms in migraine : Current knowledge and future directions. Cephalalgia 43(2):3331024221145916 10.1177/03331024221145916 36759209

[13] Ha M, Kim VN (2014). Regulation of microRNA biogenesis. Nat Rev Mol Cell Biol.

[14] Kos MZ, Puppala S, Cruz D, Neary JL, Kumar A, Dalan E (2022). Blood-Based miRNA Biomarkers as Correlates of Brain-Based miRNA Expression. Front Mol Neurosci.

[15] Buzzi MG, Moskowitz MA (2005). The pathophysiology of migraine: Year 2005. J Headache Pain.

[16] Gerring Z, Rodriguez-Acevedo AJ, Powell JE, Griffiths LR, Montgomery GW, Nyholt DR (2016). Blood gene expression studies in migraine: potential and caveats. Cephalalgia.

[17] Aczél T, Kun J, Szőke É, Rauch T, Junttila S, Gyenesei A (2018). Transcriptional alterations in the trigeminal ganglia, nucleus and peripheral blood mononuclear cells in a rat orofacial pain model. Front Mol Neurosci.

[18] Cheng CY, Chen SP, Liao YC, Fuh JL, Wang YF, Wang SJ (2018). Elevated circulating endothelial-specific microRNAs in migraine patients: a pilot study. Cephalalgia.

[19] Zhai Y, Zhu YY (2018). MiR-30a relieves migraine by degrading CALCA. Eur Rev Med Pharmacol Sci.

[20] Chen S, Chang Y, Chou C, Juan C, Lee H, Chen L (2021). Circulating microRNAs associated with reversible cerebral vasoconstriction syndrome. Ann Neurol.

[21] Greco R, de Icco R, Demartini C, Zanaboni AM, Tumelero E, Sances G (2020). Plasma levels of CGRP and expression of specific microRNAs in blood cells of episodic and chronic migraine subjects: towards the identification of a panel of peripheral biomarkers of migraine?. J Headache Pain.

[22] Chen YH, Wang H (2021). The association between migraine and depression based on miRNA biomarkers and cohort studies. Curr Med Chem.

[23] Andersen HH, Duroux M, Gazerani P (2016). Serum microRNA signatures in migraineurs during attacks and in pain-free periods. Mol Neurobiol.

[24] Gallelli L, Cione E, Peltrone F, Siviglia S, Verano A, Chirchiglia D (2019). Hsa-miR-34a-5p and hsa-miR-375 as biomarkers for monitoring the effects of drug treatment for migraine pain in children and adolescents: a pilot study. J Clin Med.

[25] Tafuri E, Santovito D, de Nardis V, Marcantonio P, Paganelli C, Affaitati G (2015). MicroRNA profiling in migraine without aura: pilot study. Ann Med.

[26] Aczél T, Benczik B, Ágg B, Körtési T, Urbán P, Bauer W (2022). Disease-and headache-specific microRNA signatures and their predicted mRNA targets in peripheral blood mononuclear cells in migraineurs: role of inflammatory signalling and oxidative stress. J Headache Pain.

[27] Pérez RF, Santamarina P, Fernández AF, Fraga MF (2019) Epigenetics and lifestyle: the impact of stress, diet, and social habits on tissue homeostasis. Epigenetics Regen 11:461-89

[28] Manokaran S, Binoy A, Bhat D, Babu S, Bhat JG, AH MR (2021) Nutrigenomics in lifestyle disorders: a review. ECS Transactions 107(1):9249

[29] Alegría-Torres JA, Baccarelli A, Bollati V (2011). Epigenetics and lifestyle. Epigenomics.

[30] Lee PH, Macfarlane DJ, Lam TH, Stewart SM (2011). Validity of the international physical activity questionnaire short form (IPAQ-SF): A systematic review. Int J Behav Nutr Phys Act.

[31] Melhado EM, Thiers Rister HL, Galego DR, de Oliveira AB, Buttarello IA, Belucio IS (2020). Allodynia in menstrually related migraine: score assessment by Allodynia Symptom Checklist (ASC-12). Headache.

[32] Kosinski M, Bayliss MS, Bjorner JB, Ware JEJ, Garber WH, Batenhorst A (2003). A six-item short-form survey for measuring headache impact: the HIT-6. Qual Life Res.

[33] Stewart WF, Lipton RB, Dowson AJ, Sawyer J (2001). Development and testing of the Migraine Disability Assessment (MIDAS) Questionnaire to assess headache-related disability. Neurology.

[34] Beck A, Steer R, Brown G, Gellman MD, Turner JR (1996). Manual for the Beck Depression Inventory-II. San Antonio Psychol Corp.

[35] Oei TPS, Evans L, Crook GM (1990). Utility and validity of the STAI with anxiety disorder patients. Br J Clin Psychol.

[36] Sanz-Carrillo C, Garcıa-Campayo J, Rubio A, Santed MA, Montoro M (2002). Validation of the Spanish version of the Perceived Stress Questionnaire. J Psychosom Res.

[37] Team RC (2013). A language and environment for statistical computing.

[38] Wickham H (2016) ggplot2: Elegant Graphics for Data Analysis. Springer-Verlag, New York. ISBN 978-3-319-24277-4. https://ggplot2.tidyverse.org

[39] Gentleman RC, et al. Bioconductor: open software development for computational biology and bioinformatics. Genome Biol. 2010;11:202. 2004;5(10):R80. 10.1186/gb-2004-5-10-r80 PMC545600 15461798

[40] Kauffmann A, Gentleman R, Huber W (2009). arrayQualityMetrics - A bioconductor package for quality assessment of microarray data. Bioinformatics.

[41] Irizarry RA, Bolstad BM, Collin F, Cope LM, Hobbs B, Speed TP (2003). Summaries of Affymetrix GeneChip probe level data. Nucleic Acids Res.

[42] Bolstad BM, Irizarry RA, Astrand M, Speed TP (2003). A comparison of normalization methods for high density oligonucleotide array data based on variance and bias. Bioinformatics.

[43] Ritchie ME, Phipson B, Wu D, Hu Y, Law CW, Shi W (2015). limma powers differential expression analyses for RNA-sequencing and microarray studies. Nucleic Acids Res.

[44] Leek JT, Johnson WE, Parker HS, Fertig EJ, Jaffe AE, Storey JD (2019). sva: Surrogate variable analysis. R package version.

[45] Lei B, Zhou J, Xuan X, Tian Z, Zhang M, Gao W (2019). Circular RNA expression profiles of peripheral blood mononuclear cells in hepatocellular carcinoma patients by sequence analysis. Cancer Med.

[46] Wang M, Liang L, Li L, Han K, Li Q, Peng Y (2017). Increased miR-424-5p expression in peripheral blood mononuclear cells from patients with pemphigus. Mol Med Rep.

[47] Smith-Vikos T, Liu Z, Parsons C, Gorospe M, Ferrucci L, Gill TM (2016). A serum miRNA profile of human longevity: findings from the Baltimore Longitudinal Study of Aging (BLSA). Aging (Albany NY).

[48] Freedman JE, Ercan B, Morin KM, Liu CT, Tamer L, Ayaz L (2012). The distribution of circulating microRNA and their relation to coronary disease. F1000Res.

[49] Benjamini Y, Yekutieli D (2001) The control of the false discovery rate in multiple testing under dependency. Ann Statist 29(4):1165–88

[50] Pérez LO, González-José R, García PP (2016). Prediction of non-genotoxic carcinogenicity based on genetic profiles of short term exposure assays. Toxicol Res.

[51] Gunther EC, Stone DJ, Gerwien RW, Bento P, Heyes MP (2003). Prediction of clinical drug efficacy by classification of drug-induced genomic expression profiles in vitro. Proc Natl Acad Sci.

[52] Man MZ, Dyson G, Johnson K, Liao B (2004). Evaluating methods for classifying expression data. J Biopharm Stat.

[53] Breiman L, Friedman JH, Olshen RA, Stone CJ (2017) Classification and regression trees. Routledge. Chapman & Hall/CRC, Boca Raton. 10.1201/9781315139470

[54] Breiman L (1996). Bagging predictors. Mach Learn.

[55] Díaz-Uriarte R, Alvarez de Andrés S (2006). Gene selection and classification of microarray data using random forest. BMC Bioinform.

[56] Diaz-Uriarte R (2007). GeneSrF and varSelRF: a web-based tool and R package for gene selection and classification using random forest. BMC Bioinform.

[57] Ru Y, Kechris KJ, Tabakoff B, Hoffman P, Radcliffe RA, Bowler R (2014). The multiMiR R package and database: Integration of microRNA-target interactions along with their disease and drug associations. Nucleic Acids Res.

[58] Ulgen E, Ozisik O, Sezerman OU (2019). pathfindR: an R package for comprehensive identification of enriched pathways in omics data through active subnetworks. Front Genet.

[59] Dogan B, Gumusoglu E, Ulgen E, Sezerman OU, Gunel T (2022). Integrated bioinformatics analysis of validated and circulating miRNAs in ovarian cancer. Genomics Inform.

[60] Mondal I, Kulshreshtha R (2021). Potential of microRNA based diagnostics and therapeutics in glioma: a patent review. Expert Opin Ther Pat.

[61] Manack AN, Buse DC, Lipton RB (2011). Chronic migraine: Epidemiology and disease burden. Curr Pain Headache Rep.

[62] Alpuente A, Gallardo VJ, Torres-Ferrús M, Álvarez-Sabin J, Pozo-Rosich P (2020). Short and mid-term predictors of response to OnabotulinumtoxinA: real-life experience observational study. Headache.

[63] Keller A, Leidinger P, Lange J, Borries A, Schroers H, Scheffler M (2009). Multiple sclerosis: microRNA expression profiles accurately differentiate patients with relapsing-remitting disease from healthy controls. PLoS ONE.

[64] Geekiyanage H, Jicha GA, Nelson PT, Chan C (2012). Blood serum miRNA: non-invasive biomarkers for Alzheimer’s disease. Exp Neurol.

[65] Schipper HM, Maes OC, Chertkow HM, Wang E (2007). MicroRNA expression in Alzheimer blood mononuclear cells. Gene Regul Syst Bio..

[66] Schroeter ML, Abdul-Khaliq H, Krebs M, Diefenbacher A, Blasig IE (2008). Serum markers support disease-specific glial pathology in major depression. J Affect Disord.

[67] Paul S, Vázquez LAB, Uribe SP, Reyes-Pérez PR, Sharma A (2020). Current status of microrna-based therapeutic approaches in neurodegenerative disorders. Cells.

[68] Chakraborty C, Sharma AR, Sharma G, Lee SS (2021). Therapeutic advances of miRNAs: a preclinical and clinical update. J Adv Res.

[69] Zhai Y, Zhu YY (2018). MiR-30a relieves migraine by degrading CALCA. Eur Rev Med Pharmacol Sci.

[70] Feil R, Fraga MF (2012). Epigenetics and the environment: emerging patterns and implications. Nat Rev Genet.

[71] Waterland RA, Jirtle RL (2003). Transposable elements: targets for early nutritional effects on epigenetic gene regulation. Mol Cell Biol.

[72] Lee KW, Pausova Z (2013). Cigarette smoking and DNA methylation. Front Genet.

[73] Ponomarev I, Wang S, Zhang L, Harris RA, Mayfield RD (2012). Gene coexpression networks in human brain identify epigenetic modifications in alcohol dependence. J Neurosci.

[74] Kamat PK, Mallonee CJ, George AK, Tyagi SC, Tyagi N (2016). Homocysteine, alcoholism, and its potential epigenetic mechanism. Alcohol Clin Exp Res.

[75] Franklin TB, Russig H, Weiss IC, Gräff J, Linder N, Michalon A (2010). Epigenetic transmission of the impact of early stress across generations. Biol Psychiatry.

[76] Wallace RG, Twomey LC, Custaud MA, Turner JD, Moyna N, Cummins PM (2018). The role of epigenetics in cardiovascular health and ageing: A focus on physical activity and nutrition. Mech Ageing Dev.

[77] Menke A, Binder EB (2022) Epigenetic alterations in depression and antidepressant treatment. Dialogues Clin Neurosci 16(3):395–404 10.31887/DCNS.2014.16.3/amenke PMC4214180 25364288

[78] Terlizzi R, Bacalini MG, Pirazzini C, Giannini G, Pierangeli G, Garagnani P (2018). Epigenetic DNA methylation changes in episodic and chronic migraine. Neurol Sci.

[79] Bocchio-Chiavetto L, Maffioletti E, Bettinsoli P, Giovannini C, Bignotti S, Tardito D (2013). Blood microRNA changes in depressed patients during antidepressant treatment. Eur Neuropsychopharmacol.

[80] Blažeković A, Borovečki F (2021). Psychiatric Comorbidities in Parkinson’s Disease Seen through the Prism of Genomics and Epigenetics. Psychiatr Danub.

[81] Citraro R, Leo A, de Caro C, Nesci V, Gallo Cantafio ME, Amodio N (2020). Effects of histone deacetylase inhibitors on the development of epilepsy and psychiatric comorbidity in WAG/Rij rats. Mol Neurobiol.

[82] Cain SM, Snutch TP (2011). Voltage-gated calcium channels and disease. BioFactors.

[83] Pietrobon D (2013). Calcium channels and migraine. Biochimica et Biophysica Acta (BBA)-Biomembranes.

[84] Anne D, Elisabeth TL, Marie-Germaine B (2002). The genetics of Migraine. Lancet Neurol.

[85] Turnpenny PD, Ellard S (2012). Alagille syndrome: pathogenesis, diagnosis and management. Eur J Hum Genet.

[86] Rubino E, Fenoglio P, Gallone S, Govone F, Vacca A, de Martino P (2013). Genetic variants in the NOTCH4 gene influence the clinical features of migraine. J Headache Pain.

[87] Vriens J, Appendino G, Nilius B (2009). Pharmacology of vanilloid transient receptor potential cation channels. Mol Pharmacol.

[88] Julius D (2013). TRP channels and pain. Annu Rev Cell Dev Biol.

[89] Jardín I, López JJ, Diez R, Sánchez-Collado J, Cantonero C, Albarrán L (2017). TRPs in pain sensation. Front Physiol.

[90] Russell FA, King R, Smillie SJ, Kodji X, Brain SD (2014). Calcitonin gene-related peptide: physiology and pathophysiology. Physiol Rev.

[91] Goadsby PJ, Edvinsson L (1993). The trigeminovascular system and migraine: studies characterizing cerebrovascular and neuropeptide changes seen in humans and cats. Ann Neurol.

[92] Tso AR, Goadsby PJ (2017). Anti-CGRP monoclonal antibodies: the next era of migraine prevention?. Curr Treat Options Neurol.

[93] Edvinsson L, Haanes KA, Warfvinge K, Krause DN (2018). CGRP as the target of new migraine therapies—successful translation from bench to clinic. Nat Rev Neurol.

[94] Charles A, Pozo-Rosich P (2019). Targeting calcitonin gene-related peptide: a new era in migraine therapy. Lancet.

[95] Benemei S, Dussor G (2019). TRP channels and migraine: recent developments and new therapeutic opportunities. Pharmaceuticals.

[96] Torres-Ferrús M, Gallardo VJ, Alpuente A, Caronna E, Gine-Cipres E, Pozo-Rosich P (2021) The impact of anti-CGRP monoclonal antibodies in resistant migraine patients: a real-world evidence observational study. J Neurol 268(10):3789–98 10.1007/s00415-021-10523-8 33772636

[97] Alpuente A, Gallardo VJ, Asskour L, Caronna E, Torres-Ferrus M, Pozo-Rosich P (2022) Salivary CGRP can monitor the different migraine phases: CGRP (in) dependent attacks. Cephalalgia 42(3):186–96 10.1177/03331024211040467 34601944

[98] Alpuente A, Gallardo VJ, Asskour L, Caronna E, Torres-Ferrus M, Pozo-Rosich P (2022) Salivary CGRP and Erenumab Treatment Response: Towards Precision Medicine in Migraine. Ann Neurol 92(5):846–59 10.1002/ana.26472 36054144

[99] Yang D gang, Gao Y yao, Yin Z qun, Wang X rui, Meng X she, Zou T feng, et al (2022) Roxadustat alleviates nitroglycerin-induced migraine in mice by regulating HIF-1α/NF-κB/inflammation pathway. Acta Pharmacol Sin (2):308–20 10.1038/s41401-022-00941-3 PMC9889379 35948752

[100] Mirouliaei M, Fallah R, Bashardoost N, Partovee M, Ordooei M (2012). Efficacy of levothyroxine in migraine headaches in children with subclinical hypothyroidism. Iran J Child Neurol.

[101] Paolucci M, Altamura C, Vernieri F (2021). The role of endothelial dysfunction in the pathophysiology and cerebrovascular effects of migraine: a narrative review. J Clin Neurol.

[102] Peres MFP, del Rio MS, Seabra ML, Tufik S, Abucham J, Cipolla-Neto J (2001). Hypothalamic involvement in chronic migraine. J Neurol Neurosurg Psychiatry..

[103] Borsook D, Erpelding N, Lebel A, Linnman C, Veggeberg R, Grant PE (2014). Sex and the migraine brain. Neurobiol Dis.

[104] MacGregor EA, Frith A, Ellis J, Aspinall L, Hackshaw A (2006). Incidence of migraine relative to menstrual cycle phases of rising and falling estrogen. Neurology.

[105] Phillips WJ, Ostrovsky O, Galli RL, Dickey S (2006). Relief of acute migraine headache with intravenous oxytocin: report of two cases. J Pain Palliat Care Pharmacother.

[106] Tzabazis A, Kori S, Mechanic J, Miller J, Pascual C, Manering N (2017). Oxytocin and migraine headache. Headache.

[107] Chandramohan R, Wu PY, Phan JH, Wang MD (2013) Benchmarking RNA-Seq quantification tools. Annu Int Conf IEEE Eng Med Biol Soc 2013:647–50. 10.1109/EMBC.2013.6609583 10.1109/EMBC.2013.6609583 PMC5003039 24109770

[108] Hayer KE, Pizarro A, Lahens NF, Hogenesch JB, Grant GR (2015). Benchmark analysis of algorithms for determining and quantifying full-length mRNA splice forms from RNA-seq data. Bioinformatics.

[109] Robinson MD, Oshlack A (2010). A scaling normalization method for differential expression analysis of RNA-seq data. Genome Biol.

[110] Rao MS, van Vleet TR, Ciurlionis R, Buck WR, Mittelstadt SW, Blomme EAG (2019). Comparison of RNA-Seq and microarray gene expression platforms for the toxicogenomic evaluation of liver from short-term rat toxicity studies. Front Genet.

[111] Kemper RHA, Meijler WJ, Korf J, ter Horst GJ (2001). Migraine and function of the immune system: a meta-analysis of clinical literature published between 1966 and 1999. Cephalalgia.

[112] Forcelini CM, Dantas DCM, Luz C, Santin R, Stein AT, Barros HMT (2011). Analysis of leukocytes in medication-overuse headache, chronic migraine, and episodic migraine. Headache.

[113] Grazzi L, Corsini E, Ciusani E, Usai S, Vasco C, Bussone G (2014). Evaluation of immune parameters in chronic migraine with medication overuse. Neurol Sci.

[114] Kok MGM, de Ronde MWJ, Moerland PD, Ruijter JM, Creemers EE, Pinto-Sietsma SJ (2018). Small sample sizes in high-throughput miRNA screens: a common pitfall for the identification of miRNA biomarkers. Biomol Detect Quantif.

